# Supplementary material for: The Isolation and Characterization of a Novel Group III-Classified Getah Virus from a Commercial Modified Live Vaccine against PRRSV
Source: Viruses. 2023 Oct 14;15(10):2090. doi: 10.3390/v15102090 (PMC10611409; doi:10.3390/v15102090)
Supplement: Supplementary file 1 [file viruses-15-02090-s001.zip › viruses-2651252-supplementary.pdf]

Supplementary Table

Table S1:

Primer sequences used in this study

Gene name	Primer sequence (5'-3')	Application
GETV-1	F: GGATCCATGGCGGACGTGTGACATC<br>R: GAATTCGTTTCTTTGCGTCCTACCGTTC	RT-PCR
GETV-2	F: GGATCCGACGGGTATACTTGCCACTGAC<br>R: GAATTCGCCACCAGATCGCGAGTC	RT-PCR
GETV-3	F: GGATCCCAGCCCCGTACCACACGACG<br>R: GAATTCGAAATGGAAACCTGGCTGCG	RT-PCR
GETV-4	F: GGATCCGCGGCCTATTCTCAGCGC<br>R: GAATTCGTAGATAACCACGTCTGCGTCG	RT-PCR
GETV-5	F: GGATCCGTCCACAGGCACATTCTCCG<br>R: GAATTCCCGAACTGTATTCCTGTGCTG	RT-PCR
GETV-6	F: GGATCCGAGTTACCGTGGGAGCCG<br>R: GAATTCCCTCCGCCGACATGTCAAAC	RT-PCR
GETV-7	F: GGATCCGCCACGGCATATTTATGCGG<br>R: GAATTCGCCGGATTCTTCTGCTTAGG	RT-PCR
GETV-8	F: GGATCCGGCAAAGCACCGAAGAAGC<br>R: GAATTCCTTTGCAGGCCTGGGTGTG	RT-PCR
GETV-9	F: GGATCCGTGTGCGCTATTCGAGGC<br>R: GAATTCCACATTCGGGATCGTTGCG	RT-PCR
GETV-10	F: GGATCCCCTGCTTTGCTGCTGCAAAC<br>R: GAATTCGCCATGCATGTCGTTTTGGC	RT-PCR
GETV-11	F: GGATCCGGAGGCAGCTGTGGACATC<br>R: GAATTCCTACGTCCCCGATGGTCTG	RT-PCR
E2	F: CGGGATCCAACATGAGTGTGAC<br>R: GGAATTCTTAAGGATACAGGCCATAGTAATACAGGATAATTTC	RT-PCR
nsp3	F: AGGGCGGTCGCTAAGAAG<br>R: TACGGTGGAGAAGTTTGGTC	RT-PCR/qPCR
N	F: AAAACCAGTCC AGAGGCAAG<br>R: CGGATCAGACGCACAGTATG	RT-PCR

Table S2:

The conserved amino acid sequence of E2 after alignment and optimization analysis

Sites	Amino acids					
1	MSVTEHFNVYK	ATKPYLAYCA	DCGDGQFCYS	PVAIEKIRDE	ASDGMIKIQV	AAQIGINKGG
61	THEHNKIRYI	AGHDMKEANR	DSLQVHTSGV	CAIRGTMGHF	IVAYCPPGDE	LKVQFQDAES
121	HTQACKVQYK	HAPAPVGREK	FTVRPHFGIE	VPCTTYQLTT	APTEEEIDMH	TPPDIPDITL
181	LSQQSGNVKI	TAGGKTIRYN	CTCGSGNVGT	TSSDKTINSC	KIAQCHAAVT	NHDKWQYTSS
241	FVPRADQLSR	KGKVHVPFPL	TNSTCRVPVA	RAPGVTYGKR	ELTVKLHPDH	PTLLTYRSLG
301	ADPRPYEEWI	DRYVERTIPV	TEDGIEYRWG	NNPPVRLWAQ	LTTEGKPHGW	PHEIILYYYG
361	LYPAATIAAV	SAAGLAVVLS	LLASCYMFAT	ARRKCLTPYA	LTPGAVVPVT	LGVLCCAPRA
421	HA*					

Notes: The strains used for alignment and optimization analysis (GenBank numbers: QFQ50494.1, QUU10704.1, QUU10669.1, QNS37105.1, QNN25872.1, QFR03303.1, QRM13508.1, QNN25874.1, AYO86696.1, QNU09889.1, QUU10668.1, QUU10667.1, AWH12890.1, AYP19283.1, ATL75445.1, AZI15597.1, AYO86694.1, ASA40294.1, ATO59745.1, ATG71347.1, BAU24798.1, ARK36626.1, ATC30860.1, ASV71654.1, ABV68939.1, ABV68933.1, ABV68927.1, ABV68931.1, ABV68937.1, ATC30859.1, ABV68932.1, ABV68935.1, ABV68928.1, AF339484).

Table S3:

GETV Strains used for sequence alignments and phylogenetic analysis in this study

Strain	GenBank number	Host	Country	Year
14-I-605-C1	LC079088	horse	Japan	2014
16-I-676	LC223132	horse	Japan	2016
12IH26	LC152056	mosquito	Japan	2012
HNJZ-S2	KY363863	pig	China	2015
GETV-GDFS9-2018	MT086509	pig	China	2018
HNJZ-S1	KY363862	pig	China	2011
HNNY-1	MG865966	pig	China	2016
JL1707	MH72225	mosquito	China	2017
HB0234	EU015062	mosquito	China	2002
GETV-V1	KY399029	pig	China	2016
YN0540	EU015063	mosquito	China	2005
-	AY702913	pig	South Korea	2004
BJ0304	OM363683	pig	China	2021
LEIV 17741 MPR	EF631999	mosquito	Mongolia	2000
MI-110-C1	LC079086	horse	Japan	1978
M1	EU015061	mosquito	China	1964
LEIV 16275 Mag	EF631998	mosquito	Russia	2000
YN12031	KY434327	mosquito	China	2012
Sagiyama virus	AB032553	mosquito	Japan	1956
M 6-Mag 132	MW410934	mosquito	Japan	1956
MM2021	MW404214	mosquito	Malaysia	1955
SD17/09	MH106780	fox/pig	China	2017
JL1808	MH722256	cattle/pig	China	2018
HuN1	MF741771	pig	China	2017

Table S4:
LC-MS/MS analysis of E2-ECD

Score	Coverage (%)	Unique Peptides	Peptides	Amino acids	MW [kDa]	calc. pI
853.26	67.92	27	27	399	44.2	8.00

Table S5:
Percent Identity (above the diagonal) and divergence (below the diagonal) of full-length nucleotide sequences of Getah virus strains

	1	2	3	4	5	6	7	8	9	10	11	12	13	14	15	16	17	18	19	20	21
1		94.8	97.4	97.4	97.6	98.7	96.4	98.1	98.7	99.3	99.0	99.1	99.0	99.0	98.9	98.9	99.1	99.0	98.8	99.1	99.1
2	5.5		95.7	95.7	95.3	95.2	94.1	95.3	95.3	95.0	94.8	94.7	94.7	94.7	94.7	94.6	94.7	94.7	94.6	94.8	94.7
3	2.7	4.5		99.9	98.1	98.0	96.7	98.1	98.0	97.7	97.5	97.4	97.4	97.3	97.4	97.3	97.4	97.4	97.2	97.5	97.5
4	2.7	4.4	0.1		98.1	98.0	96.7	98.1	98.0	97.7	97.5	97.4	97.4	97.4	97.4	97.3	97.4	97.4	97.2	97.5	97.5
5	2.4	4.9	1.9	1.9		98.3	97.7	98.3	98.3	98.0	97.7	97.6	97.6	97.6	97.6	97.5	97.6	97.6	97.5	97.7	97.7
6	1.3	5.0	2.0	2.0	1.7		97.0	98.8	99.4	99.1	98.8	98.7	98.6	98.6	98.6	98.5	98.7	98.7	98.5	98.7	98.8
7	3.7	6.2	3.4	3.4	2.3	3.1		97.0	97.0	96.7	96.4	96.3	96.3	96.3	96.3	96.2	96.3	96.3	96.1	96.3	96.4
8	1.9	4.8	1.9	1.9	1.7	1.2	3.1		98.9	98.6	98.3	98.2	98.1	98.1	98.1	98.0	98.2	98.2	97.9	98.2	98.2
9	1.3	4.9	2.0	2.0	1.7	0.6	3.1	1.1		99.1	98.8	98.7	98.6	98.6	98.6	98.5	98.7	98.7	98.5	98.8	98.8
10	0.7	5.3	2.3	2.3	2.0	0.9	3.4	1.5	0.9		99.4	99.3	99.2	99.2	99.2	99.1	99.3	99.2	99.0	99.3	99.4
11	1.0	5.5	2.6	2.6	2.3	1.2	3.7	1.8	1.2	0.6		99.0	98.9	98.9	98.9	98.9	99.0	99.1	98.8	99.1	99.1
12	0.9	5.5	2.7	2.7	2.4	1.3	3.8	1.9	1.3	0.7	1.0		99.2	99.2	99.2	99.1	99.7	99.4	99.0	99.2	99.1
13	1.0	5.5	2.7	2.7	2.4	1.4	3.8	1.9	1.4	0.8	1.1	0.8		99.9	99.6	99.9	99.2	99.2	99.5	99.2	99.0
14	1.0	5.6	2.7	2.7	2.5	1.4	3.8	1.9	1.4	0.8	1.1	0.8	0.1		99.5	99.9	99.2	99.1	99.4	99.1	99.0
15	1.1	5.5	2.7	2.7	2.4	1.4	3.8	2.0	1.4	0.8	1.1	0.8	0.4	0.5		99.5	99.2	99.1	99.7	99.1	99.0
16	1.1	5.6	2.8	2.8	2.5	1.5	3.9	2.0	1.5	0.9	1.2	0.9	0.1	0.1	0.5		99.2	99.1	99.4	99.1	98.9
17	0.9	5.5	2.7	2.6	2.4	1.3	3.8	1.8	1.3	0.7	1.0	0.3	0.8	0.8	0.8	0.8		99.5	99.1	99.2	99.1
18	1.0	5.6	2.7	2.7	2.5	1.4	3.8	1.9	1.3	0.8	0.9	0.6	0.8	0.9	0.9	0.9	0.5		99.0	99.6	99.1
19	1.2	5.7	2.9	2.9	2.6	1.6	4.0	2.1	1.6	1.0	1.3	1.0	0.5	0.6	0.3	0.6	0.9	1.0		98.9	98.8
20	0.9	5.5	2.6	2.6	2.4	1.3	3.8	1.8	1.3	0.7	0.9	0.8	0.8	0.9	0.9	0.9	0.8	0.4	1.1		99.2
21	0.9	5.5	2.6	2.6	2.4	1.2	3.8	1.8	1.2	0.6	0.9	0.9	1.0	1.0	1.0	1.1	0.9	0.9	1.2	0.8	

Notes: 1: KY399029_GETV_V1_China_2016_pig; 2: MW404214_MM2021_Malaysia_1955_mosquito; 3: AB032553_Sagiyama_virus_Japan_1956_mosq; 4: MW410934_Sagiyama_virus_Japan_1956_mosq; 5: EF631998_LEIV_16275_Mag_Russia_2000_mos; 6: EF631999_LEIV_17741_MPR_Mongolia_2000_m; 7: KY434327_YN12031_China_2012_mosquito; 8: EU015061_M1_China_1964_mosquito; 9: LC079086_MI_110_C1_Japan_1978_horse; 10: AY702913_South_Korea_2004_pig; 11: EU015063_YN0540_China_2005__mosquito; 12: KY363862_HNJZ_S1_China_2011_pig; 13: LC152056_12IH26_Japan_2012_mosquito; 14: LC079088_14_I_605_C1_Japan_2014_horse; 15: KY363863_HNJZ_S2_China_2015_pig; 16: LC223132_16_I_676_Japan_2016_horse; 17: MG865966_HNNY_1_China_2016_pig; 18: MH722255_JL1707_China_2017_mosquito; 19: MT086509_GETV_GDFS9_2018_China_2018_pig; 20: EU015062_HB0234_China_2002_mosquito; 21: OM363683_BJ0304_China_2021_pig.

Table S6:
Percent Identity (above the diagonal) and divergence (below the diagonal) of E2 amino acid sequences of Getah virus strains

	1	2	3	4	5	6	7	8	9	10	11	12	13	14	15	16	17	18	19	20	21
1		95.7	96.4	97.2	96.9	95.7	96.2	96.7	96.7	96.9	96.9	96.9	96.9	96.9	96.9	96.9	96.0	96.7	96.2	96.4	96.7
2	4.4		99.3	98.6	98.3	97.2	97.6	98.1	98.1	98.3	98.3	98.3	98.3	98.3	98.3	98.3	97.4	98.1	97.6	97.9	98.1
3	3.6	0.7		99.3	99.1	97.9	98.3	98.8	98.8	99.1	99.1	99.1	99.1	99.1	99.1	99.1	98.1	98.8	98.3	98.6	98.8
4	2.9	1.4	0.7		99.8	98.6	99.1	99.5	99.5	99.8	99.8	99.8	99.8	99.8	99.8	99.8	98.8	99.5	99.1	99.3	99.5
5	3.1	1.7	1.0	0.2		98.3	98.8	99.8	99.8	100.0	100.0	100.0	100.0	100.0	100.0	100.0	99.1	99.8	99.3	99.5	99.8
6	4.4	2.9	2.2	1.4	1.7		98.1	98.1	98.1	98.3	98.3	98.3	98.3	98.3	98.3	98.3	97.4	98.1	97.6	97.9	98.6
7	3.9	2.4	1.7	1.0	1.2	1.9		98.6	98.6	98.8	98.8	98.8	98.8	98.8	98.8	98.8	98.1	98.6	98.3	98.3	98.6
8	3.4	1.9	1.2	0.5	0.2	1.9	1.4		99.5	99.8	99.8	99.8	99.8	99.8	99.8	99.8	98.8	99.5	99.1	99.3	99.5
9	3.4	1.9	1.2	0.5	0.2	1.9	1.4	0.5		99.8	99.8	99.8	99.8	99.8	99.8	99.8	98.8	99.5	99.1	99.8	99.5
10	3.1	1.7	1.0	0.2	0.0	1.7	1.2	0.2	0.2		100.0	100.0	100.0	100.0	100.0	100.0	99.1	99.8	99.3	99.5	99.8
11	3.1	1.7	1.0	0.2	0.0	1.7	1.2	0.2	0.2	0.0		100.0	100.0	100.0	100.0	100.0	99.1	99.8	99.3	99.5	99.8
12	3.1	1.7	1.0	0.2	0.0	1.7	1.2	0.2	0.2	0.0	0.0		100.0	100.0	100.0	100.0	99.1	99.8	99.3	99.5	99.8
13	3.1	1.7	1.0	0.2	0.0	1.7	1.2	0.2	0.2	0.0	0.0	0.0		100.0	100.0	100.0	99.1	99.8	99.3	99.5	99.8
14	3.1	1.7	1.0	0.2	0.0	1.7	1.2	0.2	0.2	0.0	0.0	0.0	0.0		100.0	100.0	99.1	99.8	99.3	99.5	99.8
15	3.1	1.7	1.0	0.2	0.0	1.7	1.2	0.2	0.2	0.0	0.0	0.0	0.0	0.0		100.0	99.1	99.8	99.3	99.5	99.8
16	3.1	1.7	1.0	0.2	0.0	1.7	1.2	0.2	0.2	0.0	0.0	0.0	0.0	0.0	0.0		99.1	99.8	99.3	99.5	99.8
17	4.1	2.7	1.9	1.2	1.0	2.7	1.9	1.2	1.2	1.0	1.0	1.0	1.0	1.0	1.0	1.0		98.8	99.8	98.6	98.8
18	3.4	1.9	1.2	0.5	0.2	1.9	1.4	0.5	0.5	0.2	0.2	0.2	0.2	0.2	0.2	0.2	1.2		99.1	99.3	99.5
19	3.9	2.4	1.7	1.0	0.7	2.4	1.7	1.0	1.0	0.7	0.7	0.7	0.7	0.7	0.7	0.7	0.2	1.0		98.8	99.1
20	3.6	2.2	1.4	0.7	0.5	2.2	1.7	0.7	0.2	0.5	0.5	0.5	0.5	0.5	0.5	0.5	1.4	0.7	1.2		99.3
21	3.4	1.9	1.2	0.5	0.2	1.4	1.4	0.5	0.5	0.2	0.2	0.2	0.2	0.2	0.2	0.2	1.2	0.5	1.0	0.7	

Notes: 1: MW404214_MM2021_Malaysia_1955_mosquito; 2: AB032553_Sagiyama_virus_Japan_1956_mosquito; 3: MW410934_Sagiyama_virus_Japan_1956_mosquito; 4: EF631998_LEIV_16275_Mag_Russia_2000_mosquito; 5: EF631999_LEIV_17741_MPR_Mongolia_2000_mpsquito; 6: KY434327_YN12031_China_2012_mosquito; 7: EU015061_M1_China_1964_mosquito; 8: LC079086_MI_110_C1_Japan_1978_horse; 9: AY702913_South_Korea_2004_pig: 10: EU015063_YN0540_China_2005__mosquito; 11: KY363862_HNJZ_S1_China_2011_pig; 12: LC152056_12IH26_Japan_2012_mosquito; 13: LC079088_14_I_605_C1_Japan_2014_horse; 14: KY363863_HNJZ_S2_China_2015_pig; 15: LC223132_16_I_676_Japan_2016_horse; 16: MG865966_HNNY_1_China_2016_pig; 17: MH722255_JL1707_China_2017_mosquito; 18: MT086509_GETV_GDFS9_2018_China_2018_pig: 19: EU015062_HB0234_China_2002_mosquito; 20: OM363683_BJ0304_China_2021_pig; 21: KY399029_GETV_V1_China_2016_pig.

Table S7:

Pathogenicity of the GETV-BJ0304 strain in mice

Clinical symptoms	Distribution of the virus	Histopathology
	Serum (+)	
Weight loss (-)	Testis (+)	Cavitation and irregularity in testis (-)
Fever (-)	Brain (-)	Neuronal degeneration and inflammatory cell infiltration in brain (-)
Paralysis in pelvic limbs (-)	Lung (-)	Congestion and hemorrhage in lung (-)
Rash (-)	Spleen (-)	Intumescence in mandibular lymph node (-)
	Kidney (+)	
	Liver (-)	
	Heart (-)	

Notes: "+" means positive, "-" means negative.

Table S8:

Unique amino acid mutations in nsP3 and E2 of BJ0304 strain compared with other highly pathogenic swine GETV strains (MH106780, MH722256, MF741771, and KY399029)

Protein	Position	Mutation (aa)	Protein	Position	Mutation (aa)
nsP3	30	S/G	E2	4	E/K
	444	E/D		171	T/I

Notes: S/G represents the amino acid varied from S (other strains) to G (BJ0304).
